# Assessment of the Impact of Metals in Wild Edible Mushrooms from Dambovita County, Romania, on Human Health

**DOI:** 10.3390/foods14203540

**Published:** 2025-10-17

**Authors:** Claudia Stihi, Crinela Dumitrescu

**Affiliations:** 1 Faculty of Sciences and Arts, Valahia University of Targoviste, 13 Aleea Sinaia St., 130004 Targoviste, Romania; 2 Academy of Romanian Scientists, 3 Ilfov St., 050044 Bucharest, Romania

**Keywords:** wild mushrooms, essential minerals, toxic metals, human health risk

## Abstract

Edible wild mushrooms have considerable nutritional value, being widely used in Romania as a traditional food. Mushrooms are an important source of essential minerals for the optimal functioning of the body and can accumulate some toxic metals that affect human health, this being the reason to investigate their metal content, and the possible risks to human health associated with consuming mushrooms. Eighteen wild edible mushroom species from the forestry areas of Dâmbovița County, Romania, were analyzed for metal content using Energy-Dispersive X-ray Fluorescence with fundamental parameter methods (EDXRF-FP). The detected concentrations varied among species as follows: 6.309–88.745 mg/kg for Fe; 0.679–3.480 mg/kg for Cu; 5.115–25.942 mg/kg for Zn; 0.236–32.025 mg/kg for Mn; 0.033–4.507 mg/kg for Ni and 0.003–0.760 mg/kg for Cr. Pb and Cd were observed at low levels, with maximum concentrations of 0.886 mg/kg and 0.850 mg/kg, respectively, highlighting significant interspecific differences in metal content. The consumption of the studied mushroom species presents variable health risks associated with metal content. Adults were generally exposed to acceptable non-carcinogenic risks, although certain species possessed elevated carcinogenic risks due to Cu, Cr, and Cd. For children, non-carcinogenic risks were significant in cases of multiple species, indicating heightened vulnerability.

## 1. Introduction

Micronutrients, including both vitamins and minerals, are indispensable for maintaining the optimal physiological performance of the human body [1]. Unlike macronutrients, which can be synthesized endogenously, these essential compounds must be supplied through dietary intake [2].

Edible mushrooms not only enrich the sensory qualities of food but also represent significant dietary sources of vitamin C, B-complex vitamins (such as folates, thiamine, riboflavin, and niacin), as well as key minerals, including potassium, iron, copper, selenium, zinc, phosphorus, and magnesium [2,3,4]. Moreover, mushrooms provide an abundance of bioactive compounds and antioxidants, including proteins, β-glucan, and ergothioneine [1,5,6,7,8].

Minerals play a critical role in sustaining cognitive processes, immune function, circulatory health, and skeletal integrity [1]. Evidence from studies investigating the links between dietary patterns and cognitive decline [5,9,10,11,12,13] indicates that higher mushroom consumption is associated with beneficial effects for sustaining brain health.

In the human body, Fe, Ca, Mg, and certain trace elements (e.g., Cu, Zn, Ni) are essential minerals, yet they may become toxic when present above specific thresholds. The body is unable to store Fe efficiently and exhibits limited absorption capacity [14,15].

For adults, gastrointestinal symptoms may appear when Fe intake exceeds 20 mg/kg of body weight, with symptom severity increasing in direct relation with the ingested dose [16].

As integral components of metalloenzymes, Cu and Zn are indispensable in trace amounts for sustaining cellular physiological activity [17,18]. However, in excessive concentrations, both elements may have toxic effects in humans and animals. Acute exposure to copper salts via ingestion may induce nausea, vomiting, and ulceration of the gastrointestinal tract, while acute inhalation exposure can lead to pulmonary injury [18]. Furthermore, recent studies suggest that elevated serum copper levels may represent a risk factor for ischemic stroke and may contribute to the initiation of atherosclerotic processes [19,20].

Heavy metal contamination poses a significant global health risk. Certain metals disrupt biological functions and growth, while others accumulate in organs, contributing to serious diseases such as cancer [21]. The physiological and biochemical effects of bioaccumulation, alongside disease severity, have been characterized in [21].

Humans are exposed to heavy metals through multiple pathways: ingestion of contaminated food, inhalation of polluted air, drinking contaminated water, and dermal contact in agricultural, industrial, pharmaceutical, and residential settings [22,23]. Non-biodegradable and persistent, these metals accumulate in the body over time. While some are essential for key biochemical and physiological processes, elevated levels can exert toxic effects [24,25,26,27].

Heavy metals such as Pb, Cd, Hg, and Cr persist in the environment, and their long-term presence leads to bioaccumulation in ecosystems and represents a serious threat to said ecosystems and living organisms including humans [28,29,30].

The accumulation of metals in wild mushrooms is influenced by both environmental and biological factors. Soil composition, pH, moisture, organic matter, climate, and anthropogenic activities interact with species-specific morphology and tissue type, resulting in variable and context-dependent metal uptake [31]. Nutrient and metal content varies by species and may differ for the same species collected in different regions [32], highlighting the need for comprehensive compositional data in metals of mushrooms growing in different areas. Consequently, assessing the concentrations of both essential and potentially toxic metals in mushrooms, as well as evaluating their implications for human health, is imperative [33,34].

Wild edible mushrooms grow seasonally in areas where humidity and temperature are optimal, especially forested areas. Their unique flavor and accessibility lead to frequent worldwide consumption [35], particularly among Romania’s rural populations [36,37,38,39]. The consequences of this dietary practice for community nutrition and health remain largely unexplored.

Romania has maintained a rich heritage of wild mushroom gathering. Across the Carpathian region and nearby areas, local communities continue to engage in the collection, processing, and trade of these fungi [39].

Research studies conducted in Romania have shown that certain frequently consumed mushrooms bioaccumulate minerals from their substrate [40,41,42,43,44]. Certain studies have been conducted in the southern areas of Romania such as the counties Dâmbovița and Prahova [43,45], northwestern areas of Romania such as Sălaj County [43,46], and in Romania’s northeastern areas including the counties Iași and Suceava [32]. The effects that the consumption of wild mushrooms with accumulated metals has on human health in Romania have not yet been sufficiently studied.

A study conducted in Romania [43] investigated the accumulation of Fe, Zn, and Cu in four species (*Russula virescens*, *Russula cyanoxantha*, *Russula foetens*, and *Russula nigrescens*) from Dâmbovița County. Concentrations in dried fruiting bodies ranged from 58.8 to 340.3 mg/kg for Fe, 19.7 to 99.6 mg/kg for Zn, and 5.0 to 9.4 mg/kg for Cu, indicating a limited ability of these species to accumulate metals from the soil [43]. The study did not evaluate potential health effects [43].

The metal content (in dried fruiting bodies) of wild edible mushroom species (*Agaricus campestris*, *Boletus edulis*, *Lepiota Procera*, and *Russula cyanoxantha*) collected from Salaj County, northwestern Romania, varied as follows: 69.3 ± 17.0–148 ± 26.5 (mg/kg) for Fe; 36.1 ± 8.–76.9 ± 9.1 (mg/kg) for Cu; 67.4 ± 26.0–157 ± 24.6 (mg/kg) for Zn; 9.21 ± 2.10–15.1 ± 2.1 (mg/kg) for Mn; 0.87 ± 0.44–1.39 ± 0.39 for Ni; 1.79 ± 0.53–2.68 ± 1.36 for Cr; 0.27 ± 0.08–0.45 ± 0.18 for Pb; 0.17 ± 0.06–0.23 ± 0.04 for Cd [36]. Zavastin et al. (2018) conducted studies on the accumulation of metals in wild edible mushrooms of the species *Armillaria mellea* (collected from the Dobrovat forest area, Iasi County, Romania), *Boletus edulis* and *Cantharellus cibarius* (collected from the Poiana Stampei area, Suceava County, Romania) [32]. The detected metal concentrations (in dried fruiting bodies) were as follows: Cu (with values ranging from 15.8 ± 0.3 mg/kg to 64.1 ± 0.7 mg/kg); Fe (with values ranging from 118 ± 2 mg/kg to 222 ± 5 mg/kg); Mn (with values ranging from 26.8 ± 0.4 mg/kg to 35.8 ± 0.5 mg/kg) and Zn (with values ranging from 77.3 ± 2.4 mg/kg to 188 ± 1 mg/kg) [32].

The metal content (in dried fruiting bodies) of wild edible mushroom species (*Agaricus bisporus*, *Agaricus campestris*, *Armillaria mellea*, *Boletus edulis*, *Macrolepiota excoriate*, *Macrolepiota procera*) collected from Prahova County, Romania, varied as follows: 0.422–0.963 (mg/kg) for Cd; 0.450–1.218 (mg/kg) for Cr; 2.745 – 3.387 (mg/kg) for Cu; 0.954–1.452 (mg/kg) for Ni and 0.796–1.972 (mg/kg) for Pb [45].

This study aims to evaluate the potential human health risks associated with consuming wild mushrooms containing metals such as Fe, Cu, Zn, Mn, Ni, Cr, Pb, and Cd. We focused on species from Dâmbovița County, Romania, which are abundant and widely consumed by local communities. This approach provides a strong foundation for generating highly relevant data on potential health impacts.

Eighteen wild edible mushroom species (*Russula virescens*, *Russula cyanoxantha*, *Russula alutacea*, *Pleurotus ostreatus*, *Amanita caesarea*, *Boletus edulis*, *Macrolepiota procera*, *Cantharellus cibarius*, *Marasmius oreades*, *Russula vesca*, *Russula lutea*, *Russula aeruginea*, *Amanita rubescens*, *Hydnum repandum, Armillaria mellea*, *Lactarius volemus*, *Boletus chrysenteron*, *Boletus griseus*) were analyzed using Energy-Dispersive X-ray Fluorescence with fundamental parameter methods (EDXRF-FP) to quantify concentrations of essential and potentially toxic metals in fresh weight. The study addresses interspecific variation in metal content and contributes to our understanding of the implications of mushroom consumption for human health, particularly in terms of non-carcinogenic and carcinogenic risks.

## 2. Materials and Methods

### 2.1. Study Area

Located in the southern part of Romania, Dâmbovița County exhibits a varied landscape, from the forested northern Carpathians to the low-lying plains of the south. Dâmbovița County ranges in altitude from approximately 100 m in the southern plains to over 1800 m in the northern Carpathian highlands. This altitudinal gradient shapes variations in temperature, humidity, and vegetation, directly influencing the seasonal growth and availability of wild mushrooms.

Dâmbovița County hosts potential metal sources, including geogenic inputs from the regional lithology, industrial emissions, agricultural activities, vehicular traffic, and waste disposal sites, collectively contributing to the accumulation of metals in local wild mushroom species.

Against this general background, the mean population density (118 inhabitants/km^2^) exceeds the national average, with lower values in the southern part and higher values in the northern part. Except for major urban centers such as Târgoviște, Moreni, Pucioasa and Gaesti, cities the population is predominantly concentrated in villages across the county. For many inhabitants of Dâmbovița’s villages, gathering wild mushrooms is both a cultural habit and a means of supplementing their diet.

### 2.2. Sampling and Samples Preparation

**Figure 1:**
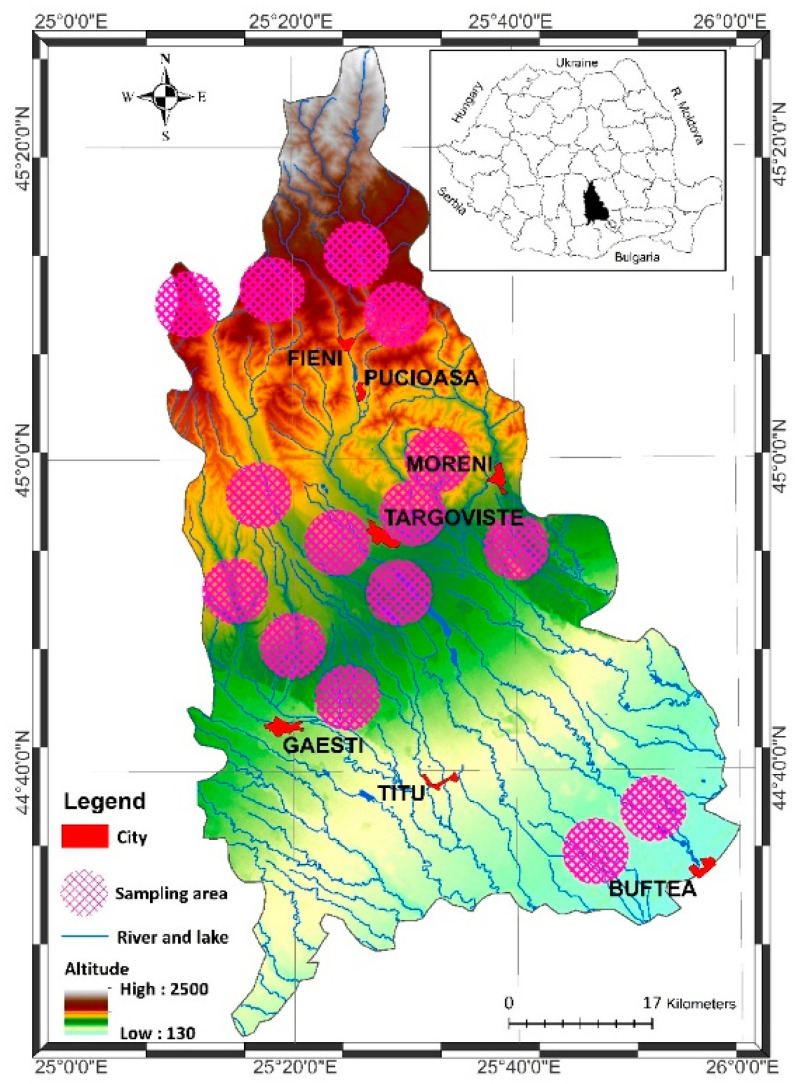
Sampling sites of wild mushrooms across Dâmbovița County, Romania.

Wild mushroom samples, corresponding to 18 edible species, with 3–5 samples for each species, were collected from 15 distinct forest ecosystems across Dâmbovița County (Figure 1) between May and October of the same year, based on their edibility, with particular attention to species traditionally harvested by local communities in the surveyed rural areas.

The Figure 1 was created using ArcGIS 10.8 software, and the Digital Terrain Model (DTM) was generated from the Shuttle Radar Topographic Mission (SRTM) data available at: https://earthexplorer.usgs.gov/. 

Healthy and mature wild mushroom samples were collected, and a preliminary cleaning step was performed to remove soil, leaf litter, and other debris without compromising the tissue structure. Each sample was assigned a unique identification code and accompanied by documentation of the sampling site, habitat and topography.

The sampled wild mushrooms were classified into three edibility categories, highly edible, generally edible, and moderately edible, reflecting both culinary value and local harvesting practices [47].

Table 1 summarizes details about the sampled wild mushrooms, including their taxonomic family, species designation, associated habitat/topography, vernacular name, and typical growth period.

The mushroom samples were transported to a laboratory in clear paper bags to minimize moisture accumulation and preserve integrity.

In the laboratory, the mushroom samples were gently rinsed with ultrapure deionized water (Millipore, Carlsbad, CA, USA), briefly air-dried to eliminate surface moisture, weighed to determine their initial fresh mass, and subsequently oven-dried at 60 °C for 48 h under controlled conditions to ensure complete dehydration.

The dried material was weighed and then finely ground using an agate mortar (Sartorius, Göttingen, Germany) and sieved to obtain a homogeneous powder. The obtained powder was pressed on Myler thin film (PREMIER Lab Supply, Port St. Lucie, FL, USA) without any chemical treatment on a Teflon sample holder and placed directly in the X-ray beam for the determination of metal content.

### 2.3. Energy-Dispersive X-Ray Fluorescence Quantitative Analysis with Fundamental Parameters Method (EDXRF-FP)

The EDXRF-FP measurements were carried out using a benchtop ElvaX Energy-Dispersive X-ray Fluorescence spectrometer (Elvatech, Kyiv, Ukraine) with an air-cooled X-ray tube featuring a rhodium cathode, a high-voltage power supply of up to 50 kV, a Si-PIN X-ray detector thermoelectrically cooled with a 165 eV energy resolution at Mn Ka and a multichannel analyzer. The system was fully controlled by a computer using the ElvaXTM 2.8 software analysis package with a data library and X-ray mathematical models.

Quantitative analysis was conducted using the fundamental parameter method [48]. Standard reference materials, NIST 1573a (tomato leaves) and NIST 1515 (apple leaves), were used to calibrate the system and to obtain the experimental parameters. A duration of 1200 s was used for X-ray spectrum acquisition in three replicates for each sample. All spectra obtained were processed to quantify the concentrations of Fe, Cu, Zn, Mn, Ni, Cr, Pb, and Cd in the wild edible mushrooms samples.

Based on instrument calibration and background signal measurements, the detection limit values for the analyzed metals in the dried mushroom samples were as follows: 2.0 mg/kg for Fe, 0.5 mg/kg for Cu, 1.0 mg/kg for Zn, 0.8 mg/kg for Mn, 0.3 mg/kg for Ni, 0.2 mg/kg for Cr, 0.1 mg/kg for Pb, and 0.05 mg/kg for Cd.

The accuracy of the method was checked by analyzing the standard reference material NIST 1575 (pine needles), and the Fe, Cu, Zn, Mn, Ni, Pb, and Cd concentrations were in good agreement, with a recovery between 87.3% and 108.2% (Table 2).

The accuracy for Cr determination was evaluated using high-purity chromium powder (≥99%, Merck, Darmstadt, Germany) as a matrix-approximated reference material. Analyses conducted under identical instrumental conditions to those used for the mushroom samples yielded a recovery of 90.2%. No recovery-based correction was applied to the reported concentration values.

Statistical analysis based on metal concentrations and mushroom species was performed using Python 3.10.9.

### 2.4. Human Health Risk Assessment

The human health risk associated with the consumption of edible wild mushrooms containing metals in different concentrations was assessed for two age groups, children and adults, following the methodology of the United States Environmental Protection Agency (USEPA) [49,50,51,52]. Non-carcinogenic risks were quantified via hazard quotients (HQs) and cumulative hazard indices (HIs), while carcinogenic risks were evaluated through incremental lifetime cancer risk (ILCR) calculations.

The Estimated Daily Intake (EDI) of Fe, Cu, Zn, Mn, Ni, Cr, Pb and Cd through wild mushroom consumption was calculated using Equation (1) [53]:

(1) EDI = CM × IR × ED × EF/BW × AT,

where EDI is the Estimated Daily Intake (mg kg^−1^day^−1^); CM is the metal concentration in wild edible mushroom samples (mg/kg fresh weight); IR is the metal intake rate (kg/day); ED is the exposure duration (years); EF is the exposure frequency (days/year); BW is the body weight (kg); AT is the average time exposure to a metal during the lifespan time (days).

The IR was assumed to be 0.3 kg/day per individual [31,54,55] while the EDI was set at 26 years for adults [56,57,58,59] and 11 years for children [52]. The EF was considered 90 days/year, reflecting the typical growth period of mushrooms and the BW was considered 70 kg for adults and 20 kg for children [60]. The AT value, in case of non-carcinogenic risk, was considered 9100 days for adults, respectively, as 4015 days for children [56,57,58,59,60].

Following USEPA guidelines, the potential health risk from metal ingestion was evaluated using HQ values and determined using Equation (2):

(2) HQ = EDI/RfD,

where RfD represents the reference dose for each metal, expressed in mg kg^−1^day^−1^ [61,62].

The HI values were determined based on the EPA guidelines for health risk assessment as the sum of the resulting HQs for each metal [61,62,63,64,65,66].

HQ and HI values below 1 denote negligible non-carcinogenic risk, while values ≥1 indicate potential for significant non-carcinogenic effects [61,65,66].

ILCR represents the estimated probability of developing cancer over a lifetime from chronic exposure to a carcinogenic substance. ILCR values in the case of metal exposure were determined using Equation (3) [62,67,68]:

(3) ILCR = ADI × CSF,

where ADI denotes the average daily intake (kg/day) of metal; CSF represents the cancer slope factor, defined as the estimated risk of developing cancer associated with a lifetime exposure to 1 mg of the metal per kilogram of body weight per day.

The total ILCR for wild mushrooms with different concentrations of metals was determined by summing the ILCR contributions of each metal. An ILCR within the range of 10^−7^ to 10^−8^ is considered minimal, values from 10^−6^ to 1 × 10^−4^ represent an acceptable level of risk, while an ILCR value exceeding 10^−4^ is indicative of a significant potential carcinogenic hazard [62,67,68].

## 3. Results and Discussion

### 3.1. Metal Content of Studied Wild Mushroom Samples

The mean concentrations of Fe, Cu, Zn, Mn, Ni, Cr, Pb, and Cd in the analyzed wild mushroom samples, determined via EDXRF-FP, are summarized in Table 3, reported on a fresh weight (fw) basis. Metal concentrations were calculated for fresh mushroom samples, reflecting typical consumption, by adjusting dry-matter EDXRF-FP measurements according to sample masses before and after drying.

The Fe concentration varied from 6.309 ± 0.525 mg/kg in *Boletus griseus* to 88.745 ± 4.337 mg/kg in *Boletus chrysenteron*; the Cu concentration ranged from 0.679 ± 0.040 in *Boletus griseus* to 3.480 ± 0.150 in *Russula lutea*; the Zn concentration ranged from 5.115 ± 0.245 mg/kg in *Lactarius volemus* to 25.942 ± 1.127 mg/kg in *Hydnum repandum*; the Mn concentration ranged from 0.236 ± 0.010 mg/kg in *Lactarius volemus* to 32.025 ± 1.800 mg/kg in *Russula alutacea*, being undetectable in *Boletus edulis*, *Russula lutea*, and *Boletus griseus*; the Ni concentration varied between 0.033 ± 0.002 mg/kg in *Russula lutea* and 4.507 ± 0.200 mg/kg in *Russula cyanoxantha*; the Cr concentration ranged from 0.003 ± 0.001 mg/kg in *Cantharellus cibarius* to 0.760 ± 0.041 mg/kg in *Boletus edulis*, being undetectable in *Macrolepiota procera* and *Russula lutea*; the Pb concentrations ranged from 0.017 ± 0.001 mg/kg in *Russula virescens* to 0.886 ± 0.040 mg/kg in *Pleurotus ostreatus*, with non-detectable levels in *Macrolepiota procera* and *Russula lutea*; and the Cd concentrations ranged from 0.012 ± 0.001 mg/kg in *Pleurotus ostreatus* to 0.850 ± 0.020 mg/kg in *Boletus chrysenteron*, being undetectable in *Russula alutacea*, *Boletus edulis*, *Macrolepiota procera*, *Cantharellus cibarius*, *Marasmius oreades*, *Russula lutea*, *Russula aeruginea* and *Russula aeruginea*.

According to the European Commission Regulation No. 915/2023, which sets maximum permissible levels of contaminants in foodstuffs, threshold values are specified only for Cd (0.5 mg/kg fw) and Pb (0.8 mg/kg fw) in mushrooms, including wild species [69]. The analytical data obtained from the studied wild mushrooms revealed that the regulatory limit was exceeded for Cd only in *Boletus chrysenteron*.

**Figure 2:**
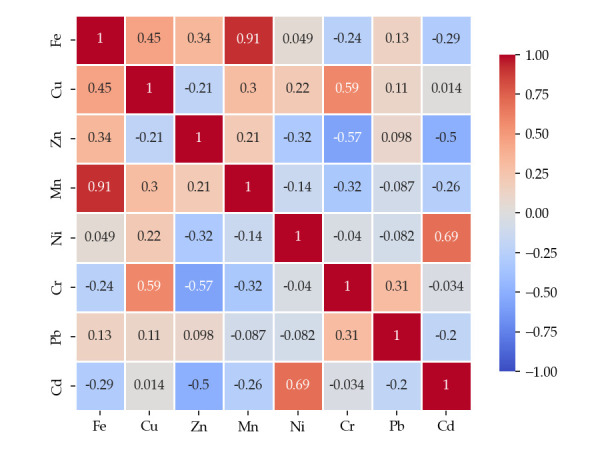
Pearson correlation matrix of metals in highly edible mushrooms, *p* < 0.05.

**Figure 3:**
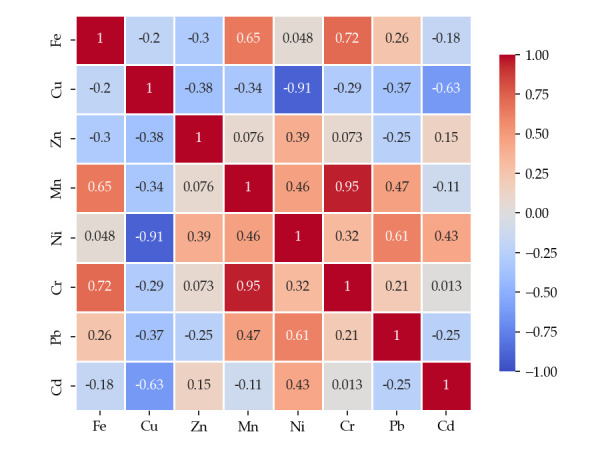
Pearson correlation matrix of metals in generally edible mushrooms, *p* < 0.05.

**Figure 4:**
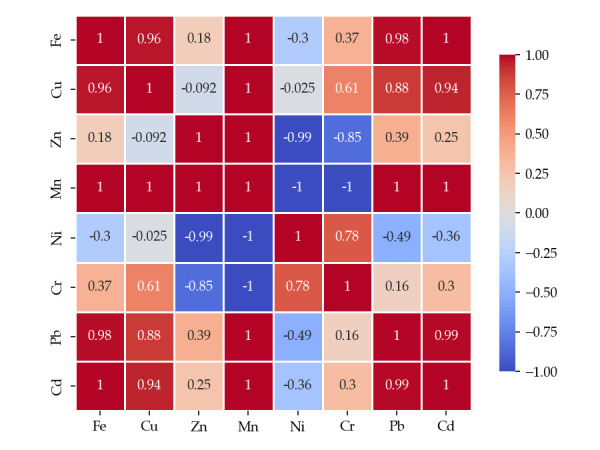
Pearson correlation matrix of metals in moderate edible mushrooms, *p* < 0.05.

To assess the relationships between metals determined in the wild mushroom samples studied, Pearson correlation analysis was performed using Python 3.10.9, and the obtained correlation matrix is presented in Figure 2, Figure 3 and Figure 4.

For highly edible mushrooms, the correlation matrix indicates strong positive associations between Fe and Mn (r = 0.91) and between Ni and Cd (r = 0.69). These strong associations suggest that the respective metal pairs may originate from comparable environmental sources or follow similar biochemical pathways during uptake. Moderate positive relationships, such as Fe–Cu (r = 0.45) and Cu–Zn (r = 0.21), indicate partial co-occurrence tendencies rather than the previously mentioned associations. In contrast, notable negative correlations, such as Cr–Cu (r = –0.59) and Cr–Zn (r = –0.57), indicate divergent accumulation routes or distinct environmental factors influencing metal bioavailability. Elements showing weak or negligible correlations, such as Pb with most other metals, likely reflect independent accumulation mechanisms or different geochemical behavior.

For generally edible mushrooms, the correlation matrix reveals several prominent patterns. Strong positive associations are observed between Cr and Zn (r = 0.95) and between Cr and Mn (r = 0.92), suggesting shared environmental sources or tightly linked uptake processes. Also, Fe demonstrates substantial correlations with Mn (r = 0.65) and Cr (r = 0.72), reflecting a tendency for co-accumulation. Conversely, Cu exhibits strong negative relationships with Ni (r = –0.91) and Cr (r = –0.79), suggesting an antagonistic interaction or contrasting geochemical behaviors. Weak or statistically insignificant correlations, such as those between Cd and most other elements, point toward independent accumulation dynamics.

The moderate edible mushroom group displays a distinct correlation pattern characterized by extremely strong positive associations among several metals. Fe shows nearly perfect correlations with Cu (r = 0.96), Pb (r = 0.98) and Cd (r = 0.94), suggesting co-mobilization and shared environmental or biological pathways of accumulation. Pb also demonstrates strong positive correlations with Cu (r = 0.88) and Cd (r = 0.99), supporting the hypothesis of a common origin or geochemical behavior. Conversely, Zn and Mn exhibit an almost perfect negative correlation (r = –0.99), while Cr–Zn (r = –0.85) shows a similar inverse trend, indicating distinct or competitive uptake mechanisms. Ni presents weak or negative correlations with most other elements, in particularly with Mn (r = –1.0), further supporting the idea of an independent accumulation pathway.

**Figure 5:**
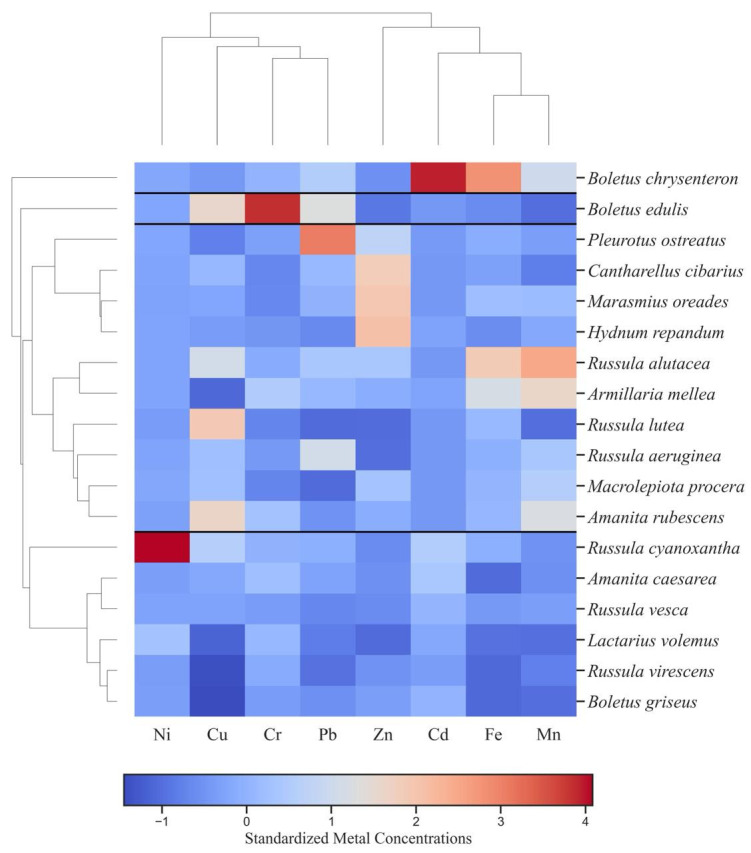
Hierarchical cluster map of studied mushrooms species.

The hierarchical cluster map presented in Figure 5 illustrates the grouping of mushroom species according to the similarity of their standardized metal concentrations [70]. Color gradients represent standardized values, where red shades denote above-mean concentrations and blue shades indicate below-mean values, while black horizontal lines delineate the distinct clusters.

Four distinct clusters of mushroom species were identified. Cluster 1, including *Russula alutacea*, *Pleurotus ostreatus*, *Macrolepiota procera*, *Cantharellus cibarius*, *Marasmius oreades*, *Russula lutea*, *Russula aeruginea*, and *Amanita rubescens*, is characterized by moderate to high Zn and Mn levels, reflecting a relatively balanced yet metal-enriched profile.

Cluster 2, comprising *Russula virescens*, *Russula cyanoxantha*, *Amanita caesarea*, *Russula vesca*, *Armillaria mellea*, and *Boletus griseus,* presents lower Fe and Pb levels alongside elevated Cu and Cd levels, suggesting species-specific differences in metal uptake or environmental sources.

Cluster 3, represented by *Boletus edulis*, is characterized by distinctly high Cr and Pb concentrations, identifying it as a specialized accumulator.

Cluster 4, consisting exclusively of *Boletus chrysenteron*, displays pronounced Fe and Mn accumulation, indicating a strong accumulation capacity for these elements. Overall, the clustering emphasizes species-specific metal accumulation patterns: *Russula* species cluster together, reflecting relatively homogeneous trace-metal profiles.

### 3.2. The Daily Metal Intake Estimated

Within the framework of the U.S. National Institutes of Health (USA NIH), the concept of Dietary Reference Intake (DRI) denotes a set of benchmark values developed to support both the evaluation and planning of nutrient intake in healthy individuals. These benchmarks, which differ with age and sex, incorporate four categories: the Recommended Dietary Allowance (RDA), Adequate Intake (AI), Estimated Average Requirement (EAR), and the Tolerable Upper Intake Level (UL) [71]. The DRIs for essential minerals such as Fe, Cu, Zn, Mn and Cr are presented in Table 4.

Daily metal intake (DMI) (mg/day) was calculated by considering the metal concentrations in studied fresh wild mushrooms samples and a consumption scenario of 300 g of fresh mushrooms per day [31,54,55]. The DMIs are summarized in Table 5 and compared with the DRIs listed in Table 4. An exceedance was observed for Fe, Cu, Mn, and Cr in certain samples.

For Fe, the DMIs for children exceeded the DRIs in the case of *Russula alutacea*, *Armillaria mellea*, and *Boletus chrysenteron*; the DMIs for male adults exceeded the DRIs in the case of *Russula cyanoxantha*, *Russula alutacea*, *Pleurotus ostreatus*, *Macrolepiota procera*, *Marasmius oreades*, *Russula lutea*, *Russula aeruginea*, *Amanita rubescens*, *Armillaria mellea* and *Boletus chrysenteron*; and the DMIs for female adults exceeded the DRIs in the case of *Russula alutacea* and *Boletus chrysenteron*. For Cu, the DMIs for children and adults (both male and female) exceeded the DRIs in the case of *Boletus edulis*, *Russula lutea* and *Amanita rubescens*. For Mn, the DMIs for male adults exceeded the DRIs in the case of *Russula alutacea*, *Macrolepiota procera*, *Marasmius oreades*, *Russula aeruginea*, *Amanita rubescens*, *Armillaria mellea*, *Boletus chrysenteron*; the DMIs for children exceeded the DRIs in the case of *Russula alutacea*, *Macrolepiota procera*, *Marasmius oreades*, *Russula aeruginea*, *Amanita rubescens*, *Hydnum repandum*, *Armillaria mellea* and *Boletus chrysenteron*; and the DMIs for female adults exceeded the DRIs in the case of *Russula alutacea*, *Pleurotus ostreatus*, *Macrolepiota procera*, *Marasmius oreades*, *Russula vesca*, *Russula aeruginea*, *Amanita rubescens*, *Hydnum repandum*, *Armillaria mellea* and *Boletus chrysenteron*. For Cr, the DMIs for men and children exceeded the DRIs in the case of *Amanita caesarea*, *Amanita rubescens*, *Armillaria mellea*, *Lactarius volemus*, *Boletus chrysenteron* and the DMIs for women exceeded the DRIs in the case of *Russula virescens*, *Russula Cyanoxantha*, *Russula alutacea*, *Amanita caesarea*, *Amanita rubescens*, *Armillaria mellea*, *Lactarius volemus*, *Boletus chrysenteron*.

For Zn, the DMIs remained consistently below the DRI thresholds, representing no more than 70.75% of the DRIs for children and male adults and up to 97.28% for female adults, indicating that Zn does not induce a nutritional excess risk according to the studied samples.

### 3.3. Non-Carcinogenic Risk Assessment

Most culinary preparations involving mushrooms require cooking, yet it remains uncertain whether thermal treatment and pH fluctuations can drive the interconversion of metal species, as this aspect has received limited scientific attention to date [73,74]. For this reason, such effects were not considered in the present study.

In this study, the EDI values were determined using Equation (1). The mean concentrations of metals obtained in the studied fresh wild mushrooms samples, assuming a daily consumption of 300 g of fresh mushrooms, like in other studies [31,54,55], were used. Also, the EF of 90 days per year was used, reflecting the typical growth season of the wild mushrooms in Romania.

The EDI values estimated for adults are reported in Table 6, whereas those for children are present in Table 7.

According to USEPA guidelines and the relevant literature [31,56,75,76,77,78,79,80,81,82], the RfD and CSF values for Fe, Cu, Zn, Mn, Ni, Cr, Pb and Cd, presented in Table 8, were used for non-carcinogenic and carcinogenic risk assessment.

The exceedances of the DRIs for Fe, Cu, Mn, and Cr, in multiple cases, among the studied wild mushrooms, indicate a potential toxicological risk for both children and adults. To evaluate this risk, HQ and HI values were determined, and they are summarized for adults in Table 9 and for children in Table 10.

For adults, all calculated HQ and HI values were below the threshold of 1, indicating the absence of significant non-carcinogenic risks associated with the consumption of wild mushrooms contaminated with metals.

For children, the HQ values consistently below 1, except for Cd in the sample of *Boletus chrysenteron*; however, the calculated HI values exceeded the safety threshold of 1 in several samples, including *Russula cyanoxantha*, *Russula alutacea*, *Pleurotus ostreatus*, *Amanita caesarea*, *Boletus edulis*, *Russula aeruginea*, *Amanita rubescens*, *Armillaria mellea* and *Boletus chrysenteron*.

These results suggest that the consumption of the analyzed wild mushroom species presents significant non-carcinogenic risks for children. The higher HI values estimated for children primarily reflect their lower body weight and higher food intake per unit of body mass, which lead to proportionally greater exposure to metals compared to adults.

The relative magnitude of risk among the species was in the following order: *Russula aeruginea* < *Amanita caesarea* < *Amanita rubescens* < *Pleurotus ostreatus* < *Armillaria mellea* < *Boletus edulis* < *Russula alutacea* < *Russula cyanoxantha* < *Boletus chrysenteron*.

### 3.4. Carcinogenic Risk Assessment

Table 11 summarizes the ILCR values calculated for adults and children based on the ADI values (Supplementary Material, Tables S1 and S2) on a fresh weight basis for the studied wild mushrooms containing Cu, Cr Pb, and Cd.

Data analysis showed that the ILCR for Cu exceeded 10^−4^ across all samples, while ILCR values for Cr surpassed this threshold for the samples of *Russula virescens*, *Russula cyanoxantha*, *Russula alutacea*, *Pleurotus ostreatus*, *Amanita caesarea*, *Boletus edulis*, *Russula vesca*, *Amanita rubescens*, *Lactarius volemus*, *Boletus chrysenteron* and *Boletus griseus*. For Cd, ILCR values above 10^−4^ were observed for the samples of *Russula virescens*, *Russula cyanoxantha*, *Pleurotus ostreatus*, *Amanita caesarea*, *Russula vesca*, *Hydnum repandum*, *Armillaria mellea*, *Lactarius volemus*, *Boletus chrysenteron* and *Boletus griseus*.

These results highlight a notable carcinogenic risk for adults consuming the studied wild mushrooms species (as indicated by the sample IDs), attributable to their Cu, Cr, and Cd content and slope factor variability.

An acceptable carcinogenic risk was suggested by the ILCR values for adults consuming wild mushrooms species corresponding to the *Cantharellus cibarius*, *Marasmius oreades*, *Russula aeruginea* and *Hydnum repandum* samples, due to the Cr content.

ILCR values for Pb, ranging from 9.06 × 10^−6^ to 1.12 × 10^−5^, indicated an acceptable carcinogenic risk for adults consuming all the studied wild mushrooms species, with the exception of the species corresponding to the *Russula virescens* sample, for which the ILCR (6.2 × 10^−7^) showed a negligible risk.

The ILCR values for Cu, Cr, Pb, and Cd associated with children’s intake exceeded 10^−4^ in the majority of the analyzed wild mushroom samples. In detail, the ILCR for Cu ranged from 3.14 × 10^−2^ (*Boletus griseus*) to 16.13 × 10^−2^ (*Russula lutea*); for Cd, it ranged from 12.03 × 10^−4^ (*Pleurotus ostreatus*) to 14.60 × 10^−2^ (*Boletus chrysenteron*); for Cr, it ranged from 5.45 × 10^−4^ (*Hydnum repandum*) to 1.03 × 10^−2^ (*Boletus edulis*); and for Pb, it varied from 2.05 × 10^−4^ (*Pleurotus ostreatus*) to 1.18 × 10^−4^ (*Boletus edulis*), and 1.07 × 10^−4^ (*Russula aeruginea*).

These results demonstrate that the ingestion of the studied edible wild mushroom species represents a substantial carcinogenic risk for children.

However, most of the ILCR values for Pb (excepting *Pleurotus ostreatus*, *Boletus edulis* and *Russula aeruginea* samples) and the ILCR for Cr in *Cantharellus cibarius* and *Marasmius oreades* remained within the 10^−6^–10^−4^ range, indicating an acceptable level of carcinogenic risk for children.

Regarding the total ILCR, values exceeded 10^−4^ for both adults and children (Table 11), indicating a substantial carcinogenic risk. Consequently, there is a clear need to implement awareness campaigns targeting rural populations to inform them of the potential health risks associated with the consumption of edible wild mushrooms, as well as to conduct surveys on consumption frequency and the specific mushroom species that can be commonly consumed.

The consumption of the studied mushroom species presents variable health risks associated with metal content. Adults were generally exposed to acceptable non-carcinogenic risks, although certain species possessed elevated carcinogenic risks due to Cu, Cr, and Cd. For children, non-carcinogenic risks were significant in multiple species, indicating heightened vulnerability.

The obtained results of this study support several recommendations aimed at preventing the accumulation of metals in wild mushrooms and minimizing the associated health risks for consumers. These include identifying and characterizing the main sources of metal pollution within the investigated area; systematically monitoring metal concentrations in soils; implementing public awareness campaigns to inform local communities about the potential health hazards linked to the consumption of wild mushrooms; and discouraging the consumption of wild edible mushrooms, particularly among children.

## 4. Conclusions

Certain metals are indispensable micronutrients for the human body, yet only when present at trace levels. Once their intake exceeds physiological requirements, they may become toxic and pose significant risks to human health.

The present study revealed that several species (*Russula virescens*, *Russula cyanoxantha*, *Russula alutacea*, *Pleurotus ostreatus*, *Amanita caesarea*, *Boletus edulis*, *Macrolepiota procera*, *Cantharellus cibarius*, *Marasmius oreades*, *Russula vesca*, *Russula lutea*, *Russula aeruginea*, *Amanita rubescens*, *Hydnum repandum*, *Armillaria mellea*, *Lactarius volemus*, *Boletus chrysenteron*, *Boletus griseus*) of wild mushrooms collected in Dâmbovița County contain metal concentrations capable of exceeding dietary safety thresholds, particularly regarding children’s exposure. Although certain metals such as Fe, Cu, and Mn are essential in trace amounts, their elevated intake through mushroom consumption may translate into significant non-carcinogenic and carcinogenic risks. While adults generally remain within safe thresholds for non-carcinogenic exposure, the elevated hazard indices and ILCR values observed in multiple species indicate that some wild mushrooms could represent a serious health concern if consumed frequently.

The obtained results highlight the necessity of incorporating wild mushroom consumption into risk–benefit dietary evaluations and reinforce the importance of continuous monitoring to safeguard public health.

Adults were generally exposed to acceptable non-carcinogenic risks, although certain species possessed elevated carcinogenic risks due to Cu, Cr, and Cd. For children, non-carcinogenic risks were significant in cases of multiple species, indicating heightened vulnerability. The results emphasize the need for the careful monitoring of metal accumulation and content in wild mushrooms to reduce both non-carcinogenic and carcinogenic health hazards. In this regard, we recommend systematically monitoring metal concentrations in soils; the implementation of public awareness campaigns to inform local communities about the potential health hazards linked to the consumption of wild mushrooms; and discouragement of the consumption of wild edible mushrooms, particularly among children.

## Figures and Tables

**Table 1:** Characteristics of wild mushrooms sampled in Dâmbovița County.

Sample ID	Taxonomic Family	Species	Associated Habitat/Topography	Vernacular Name (Romanian/ English)	Growth Period
Highly Edible					
WM1	Russulaceae	*Russula* *virescens*	Beech and mixed forests/hills, mountains	Hulubite/ Dove Mushroom	Summer–Autumn
WM2	Russulaceae	*Russula* *cyanoxantha*	Mixed deciduous and coniferous forests, clearings/hills, mountains, meadows, and gardens in plains	Vinetica porumbeilor/ Pigeon Mushroom	Summer–Autumn
WM3	Russulaceae	*Russula* *alutacea*	Deciduous forests, calcareous soils/hills, mountains	Painisoara/ Fairy Ring Mushroom	Summer–Autumn
WM4	Pleurotaceae	*Pleurotus* *ostreatus*	On deciduous trunks/plains, hills, mountains	Pastrav de fag/Beech Salmon Mushroom	Autumn–Winter
WM5	Amanitaceae	*Amanita* *caesarea*	Deciduous forests, sandy or gravelly soils/plains, mountains	Buretele domnesc/Royal Bolete	Summer–Autumn
WM6	Boletaceae	*Boletus edulis*	Deciduous forests from sunny areas/hills, mountains	Manatarca pietroasa/Boletes/Stone Mushroom	Summer–Autumn
WM7	Lepiotaceae	*Macrolepiota procera*	Deciduous forest clearings; sandy or gravelly soils/plains, hills, mountains	Palaria sarpelui/Snake’s Cap	Summer–Early Autumn
WM8	Cantharellaceae	*Cantharellus cibarius*	Coniferous and deciduous forests; among moss or leaf litter/mostly in mountains	Bureti galbeni/Chanterelle	Late Spring– Autumn
WM9	Marasmiaceae	*Marasmius oreades*	Meadows, clearings; sandy soils/along roads	Ghebe de lunca/Meadow Mushroom	Spring– Autumn
Generally Edible					
WM10	Russulaceae	*Russula vesca*	Deciduous and coniferous forests/hills and mountains	Painea pamantului/Earth Bread	Summer
WM11	Russulaceae	*Russula lutea*	Deciduous and coniferous forests/hills and mountains	Burete galben/Yellow Mushroom	Summer–Early Autumn
WM12	Russulaceae	*Russula aeruginea*	Coniferous and deciduous forests, especially under birch/plains, hills, mountains	Vinetica porcului/Piglet Mushroom	Summer–Autumn
WM13	Amanitaceae	*Amanita rubescens*	Deciduous and coniferous forests/plains, hills, mountains	Buretele rosu brobonat/Red-staining Mushroom	Spring–Autumn
WM14	Hydnaceae	*Hydnum repandum*	Deciduous and coniferous forests; hills, mountains	Flocoșel/Hedgehog Mushroom	Late Summer–Autumn
WM15	Physalacriaceae	*Armillaria mellea*	Decaying deciduous and coniferous trunks/hills, mountains	Ghebe/Honey Fungus	Autumn–Late Autumn
Moderately Edible					
WM16	Russulaceae	*Lactarius volemus*	Deciduous and coniferous forests/mountains	Laptuca dulce/Sweet Milkcap	Summer
WM17	Boletaceae	*Boletus* *chrysenteron*	Deciduous forests/mossy areas	Hribul de muschi/Moss Bolete	Summer–Autumn
WM18	Boletaceae	*Boletus griseus*	Deciduous forests/plains, hills, mountains	Buretele cenusiu/Gray Bolete	Summer–Early Autumn

**Table 2:** EDXRF-FP accuracy assessment based on recovery analysis.

Metals	SRM * Concentration (mg/kg dw **)	Measured Concentration (mg/kg dw **)	Recovery (%)
Fe	46.00 ± 2.00	40.15 ± 1.80	87.3
Cu	2.80 ± 0.20	2.89 ± 0.35	103.5
Zn	38.00 ± 2.00	34.99 ± 2.50	92.1
Mn	488.00 ± 12.00	466.04 ± 17.40	95.5
Ni	1.47 ± 0.10	1.46 ± 0.25	99.7
Cr	***	6.20 ± 0.72	Nd ****
Pb	0.167 ± 0.015	0.179 ± 0.021	105.3
Cd	0.233 ± 0.004	0.252 ± 0.035	108.2

* Standard reference material; ** dry weight; *** Cr concentration value is not included in the certificate of analysis; **** not determined.

**Table 3:** Mean concentrations of metals in studied wild mushrooms (in mg/kg fw).

Sample ID	Fe	Cu	Zn	Mn	Ni	Cr	Pb	Cd
WM1	6.503 ± 0.055	0.718 ± 0.052	8.728 ± 0.070	2.219 ± 0.020	0.061 ± 0.008	0.094 ± 0.007	0.017 ± 0.001	0.017 ± 0.001
WM2	28.289 ± 1.815	2.385 ± 0.151	8.004 ± 0.350	4.507 ± 0.335	4.507 ± 0.200	0.115 ± 0.006	0.214 ± 0.020	0.187 ± 0.007
WM3	68.996 ± 4.450	2.805 ± 0.120	14.675 ± 0.520	32.025 ± 1.800	0.163 ± 0.007	0.095 ± 0.006	0.310 ± 0.010	nd **
WM4	27.160 ± 2.318	1.252 ± 0.052	17.128 ± 0.761	6.137 ± 0.290	0.187 ± 0.007	0.066 ± 0.004	0.886 ± 0.040	0.012 ± 0.001
WM5	6.946 ± 0.447	1.731 ± 0.077	8.446 ± 0.382	4.146 ± 0.200	0.069 ± 0.002	0.155 ± 0.009	0.167 ± 0.007	0.167 ± 0.005
WM6	16.245 ± 1.722	3.205 ± 0.240	6.311 ± 0.300	nd **	0.182 ± 0.008	0.760 ± 0.041	0.507 ± 0.020	nd **
WM7	29.975 ± 2.500	2.088 ± 0.104	14.183 ± 0.950	14.549 ± 0.620	0.196 ± 0.009	nd **	nd **	nd **
WM8	22.497 ± 2.100	1.968 ± 0.085	24.281 ± 1.140	2.018 ± 0.100	0.151 ± 0.007	0.003 ± 0.001	0.249 ± 0.010	nd **
WM9	33.661 ± 1.968	1.691 ± 0.082	25.114 ± 1.051	10.737 ± 0.650	0.141 ± 0.006	0.006 ± 0.002	0.216 ± 0.009	nd **
WM10	20.340 ± 1.732	1.656 ± 0.071	8.086 ± 0.370	6.207 ± 0.220	0.139 ± 0.006	0.053 ± 0.002	0.077 ± 0.005	0.090 ± 0.006
WM11	31.624 ± 1.500	3.480 ± 0.150	5.296 ± 0.200	nd **	0.033 ± 0.002	nd **	nd **	nd **
WM12	27.536 ± 1.700	2.075 ± 0.120	5.423 ± 0.300	12.587 ± 0.550	0.158 ± 0.007	0.045 ± 0.002	0.461 ± 0.015	nd **
WM13	30.891 ± 1.940	3.245 ± 0.150	11.529 ± 0.460	20.595 ± 1.120	0.092 ± 0.006	0.166 ± 0.008	0.113 ± 0.008	nd **
WM14	16.742 ± 1.270	1.567 ± 0.085	25.942 ± 1.127	7.609 ± 0.340	0.157 ± 0.007	0.040 ± 0.001	0.089 ± 0.005	0.033 ± 0.001
WM15	54.573 ± 2.290	0.979 ± 0.050	11.442 ± 0.620	23.954 ± 2.100	0.165 ± 0.007	0.202 ± 0.015	0.247 ± 0.018	0.038 ± 0.001
WM16	8.871 ± 0.350	0.942 ± 0.032	5.115 ± 0.245	0.236 ± 0.010	0.700 ± 0.030	0.134 ± 0.008	0.050 ± 0.002	0.048 ± 0.002
WM17	88.745 ± 4.337	1.539 ± 0.080	8.405 ± 0.400	18.511 ± 0.870	0.200 ± 0.009	0.119 ± 0.005	0.344 ± 0.012	0.850 ± 0.020
WM18	6.309 ± 0.525	0.679 ± 0.040	9.914 ± 0.520	nd **	0.071 ± 0.005	0.053 ± 0.004	0.108 ± 0.003	0.088 ± 0.002

** not detected.

**Table 4:** DRIs for essential minerals [72].

Elements	Children ≥ 4 Years	Male	Female	Pregnant Women
Fe (mg/day)	10–11	8	18	27
Cu (mg/day)	0.440–0.890	0.900	0.900	1
Zn (mg/day)	5–11	11	8	11
Mn (mg/day)	1.5–2.2	2.3	1.8	2
Cr (mg/day)	0.015–0.035	0.035	0.025	0.030

**Table 5:** The DMI (mg/day) calculated for the consumption of 300 g of fresh wild mushrooms per day.

Sample ID	Fe (mg/day)	Cu (mg/day)	Zn (mg/day)	Mn (mg/day)	Ni (mg/day)	Cr (mg/day)	Pb (mg/day)	Cd (mg/day)
WM1	1.950	0.215	2.618	0.665	0.018	0.028	0.005	0.005
WM2	8.486	0.715	2.401	1.352	1.352	0.034	0.064	0.056
WM3	20.698	0.841	4.402	9.607	0.048	0.028	0.093	na *
WM4	8.148	0.375	5.138	1.841	0.056	0.019	0.265	0.002
WM5	2.083	0.519	2.533	1.243	0.020	0.046	0.050	0.050
WM6	4.873	0.961	1.893	na *	0.054	0.228	0.152	na *
WM7	8.992	0.626	4.254	4.364	0.058	na *	na *	na *
WM8	6.749	0.590	7.284	0.605	0.045	0.000	0.074	na *
WM9	10.098	0.507	7.534	3.221	0.042	0.001	0.064	na *
WM10	6.102	0.496	2.425	1.862	0.041	0.015	0.023	0.027
WM11	9.487	1.044	1.588	na *	0.009	na *	na *	na *
WM12	8.260	0.622	1.626	3.776	0.047	0.013	0.138	na *
WM13	9.267	0.973	3.458	6.178	0.027	0.049	0.033	na *
WM14	5.022	0.470	7.782	2.282	0.047	0.012	0.026	0.009
WM15	16.371	0.293	3.432	7.186	0.049	0.060	0.074	0.011
WM16	2.661	0.282	1.534	0.070	0.210	0.040	0.015	0.014
WM17	26.623	0.461	2.521	5.553	0.060	0.035	0.103	0.255
WM18	1.892	0.203	2.974	na *	0.021	0.015	0.032	0.026

* not applicable.

**Table 6:** The estimated daily intake (mg kg^−1^day^−1^) values for adults.

Sample	EDI (mg kg^−1^day^−1^)							
	Fe	Cu	Zn	Mn	Ni	Cr	Pb	Cd
*Russula virescens*	0.002	<10^−3^	0.003	<10^−3^	<10^−3^	<10^−3^	<10^−3^	<10^−3^
*Russula cyanoxantha*	0.011	<10^−3^	0.003	0.001	0.001	<10^−3^	<10^−3^	<10^−3^
*Russula alutacea*	0.026	0.001	0.005	0.012	<10^−3^	<10^−3^	<10^−3^	na *
*Pleurotus ostreatus*	0.010	<10^−3^	0.006	0.002	<10^−3^	<10^−3^	<10^−3^	<10^−3^
*Amanita caesarea*	0.002	<10^−3^	0.003	0.001	<10^−3^	<10^−3^	<10^−3^	<10^−3^
*Boletus edulis*	0.006	0.001	0.002	na *	<10^−3^	<10^−3^	<10^−3^	na *
*Macrolepiota procera*	0.011	<10^−3^	0.005	0.005	<10^−3^	na *	na *	na *
*Cantharellus cibarius*	0.008	<10^−3^	0.009	<10^−3^	<10^−3^	<10^−3^	<10^−3^	na *
*Marasmius oreades*	0.013	<10^−3^	0.009	0.004	<10^−3^	<10^−3^	<10^−3^	na *
*Russula vesca*	0.007	<10^−3^	0.003	0.002	<10^−3^	<10^−3^	<10^−3^	<10^−3^
*Russula lutea*	0.012	0.001	0.002	na *	<10^−3^	na *	na *	na *
*Russula aeruginea*	0.010	<10^−3^	0.002	0.004	<10^−3^	<10^−3^	<10^−3^	na *
*Amanita rubescens*	0.012	0.001	0.004	0.008	<10^−3^	<10^−3^	<10^−3^	na *
*Hydnum repandum*	0.006	<10^−3^	0.010	0.002	<10^−3^	<10^−3^	<10^−3^	<10^−3^
*Armillaria mellea*	0.021	<10^−3^	0.004	0.009	<10^−3^	<10^−3^	<10^−3^	<10^−3^
*Lactarius volemus*	0.003	<10^−3^	0.001	<10^−3^	<10^−3^	<10^−3^	<10^−3^	<10^−3^
*Boletus chrysenteron*	0.034	<10^−3^	0.003	0.007	<10^−3^	<10^−3^	<10^−3^	<10^−3^
*Boletus griseus*	0.002	<10^−3^	0.003	na *	<10^−3^	<10^−3^	<10^−3^	<10^−3^

* not applicable.

**Table 7:** The estimated daily intake (mg kg^−1^day^−1^) values for children.

Sample	EDI (mg kg^−1^day^−1^)							
	Fe	Cu	Zn	Mn	Ni	Cr	Pb	Cd
*Russula virescens*	0.024	0.002	0.032	0.008	<10^−3^	<10^−3^	<10^−3^	<10^−3^
*Russula cyanoxantha*	0.104	0.008	0.029	0.016	<10^−3^	<10^−3^	<10^−3^	<10^−3^
*Russula alutacea*	0.255	0.010	0.054	0.118	<10^−3^	<10^−3^	0.001	0
*Pleurotus ostreatus*	0.100	0.004	0.063	0.022	<10^−3^	<10^−3^	0.003	0
*Amanita caesarea*	0.025	0.006	0.031	0.015	<10^−3^	<10^−3^	<10^−3^	<10^−3^
*Boletus edulis*	0.060	0.011	0.023	na *	<10^−3^	0.002	0.001	na *
*Macrolepiota procera*	0.110	0.007	0.052	0.053	<10^−3^	na *	na *	na *
*Cantharellus cibarius*	0.083	0.007	0.089	0.007	<10^−3^	na *	<10^−3^	na *
*Marasmius oreades*	0.124	0.006	0.092	0.039	<10^−3^	na *	<10^−3^	na *
*Russula vesca*	0.075	0.006	0.029	0.022	<10^−3^	<10^−3^	<10^−3^	<10^−3^
*Russula lutea*	0.116	0.012	0.019	na *	<10^−3^	na *	na *	na *
*Russula aeruginea*	0.101	0.007	0.020	0.046	<10^−3^	<10^−3^	0.001	na *
*Amanita rubescens*	0.114	0.012	0.042	0.076	<10^−3^	<10^−3^	<10^−3^	na *
*Hydnum repandum*	0.061	0.005	0.095	0.028	<10^−3^	<10^−3^	<10^−3^	<10^−3^
*Armillaria mellea*	0.201	0.003	0.042	0.088	<10^−3^	<10^−3^	<10^−3^	<10^−3^
*Lactarius volemus*	0.032	0.003	0.018	<10^−3^	0.002	<10^−3^	<10^−3^	<10^−3^
*Boletus chrysenteron*	0.328	0.005	0.031	0.068	<10^−3^	<10^−3^	0.001	0.003
*Boletus griseus*	0.023	0.002	0.036	na *	<10^−3^	<10^−3^	<10^−3^	<10^−3^

* not applicable.

**Table 8:** RfD and CSF used values for non-carcinogenic and carcinogenic risk assessment [31,56,75,76,77,78,79,80,81,82].

	Fe	Cu	Zn	Mn	Ni	Cr	Pb	Cd
RfD (mg kg^−1^day^−1^)	0.7	0.04	0.3	0.14	0.02	0.003	0.0035	0.001
CSF (mg kg^−1^day^−1^)	-	1.7	-	-	not established *	0.5	0.0085	6.3

* US Environmental Protection Agency. Integrated Risk Information System (IRIS), Nickel, Soluble Salts [78].

**Table 9:** The HQ and HI values determined for adults.

Sample ID	HQ							HI
	Fe	Cu	Mn	Ni	Cr	Pb	Cd	
WM1	0.00	0.01	0.01	0.00	0.01	0.00	0.01	0.04
WM2	0.02	0.02	0.01	0.09	0.01	0.02	0.07	0.24
WM3	0.04	0.03	0.09	0.00	0.01	0.03	na *	0.20
WM4	0.02	0.01	0.02	0.00	0.01	0.10	0.00	0.16
WM5	0.00	0.02	0.01	0.00	0.02	0.02	0.07	0.14
WM6	0.01	0.03	na *	0.00	0.10	0.06	na *	0.20
WM7	0.02	0.02	0.04	0.00	na *	na *	na *	0.08
WM8	0.01	0.02	0.01	0.00	0.00	0.03	na *	0.07
WM9	0.02	0.02	0.03	0.00	0.00	0.02	na *	0.09
WM10	0.01	0.02	0.02	0.00	0.01	0.01	0.04	0.11
WM11	0.02	0.03	na *	0.00	na *	na *	na *	0.05
WM12	0.02	0.02	0.04	0.00	0.01	0.05	na *	0.14
WM13	0.02	0.03	0.06	0.00	0.02	0.01	na *	0.14
WM14	0.01	0.02	0.02	0.00	0.01	0.01	0.01	0.08
WM15	0.03	0.01	0.07	0.00	0.03	0.03	0.01	0.18
WM16	0.00	0.01	0.00	0.01	0.02	0.01	0.02	0.07
WM17	0.05	0.02	0.05	<10^−2^	0.02	0.04	0.33	0.51
WM18	<10^−2^	0.01	na *	<10^−2^	0.01	0.01	0.03	0.06

* not applicable.

**Table 10:** The HQ and HI values determined for children.

Sample ID	HQ							HI
	Fe	Cu	Mn	Ni	Cr	Pb	Cd	
WM1	0.03	0.07	0.06	0.01	0.12	0.02	0.06	0.37
WM2	0.15	0.22	0.12	0.83	0.14	0.23	0.69	2.38
WM3	0.36	0.26	0.85	0.03	0.12	0.33	na *	1.95
WM4	0.14	0.12	0.16	0.03	0.08	0.94	0.03	1.5
WM5	0.04	0.16	0.11	0.01	0.19	0.18	0.62	1.31
WM6	0.09	0.30	na *	0.03	0.94	0.54	na *	1.9
WM7	0.16	0.19	0.38	0.04	na *	na *	na *	0.77
WM8	0.12	0.18	0.05	0.03	0.00	0.26	na *	0.64
WM9	0.18	0.16	0.28	0.03	0.01	0.23	na *	0.89
WM10	0.11	0.15	0.16	0.03	0.07	0.08	0.33	0.93
WM11	0.17	0.32	na *	0.01	na *	na *	na *	0.5
WM12	0.15	0.19	0.33	0.03	0.06	0.49	na *	1.25
WM13	0.16	0.30	0.54	0.02	0.20	0.12	na *	1.34
WM14	0.09	0.14	0.20	0.03	0.05	0.09	0.12	0.72
WM15	0.29	0.09	0.63	0.03	0.25	0.26	0.14	1.69
WM16	0.05	0.09	0.01	0.13	0.17	0.05	0.18	0.68
WM17	0.47	0.14	0.49	0.04	0.15	0.36	3.14	4.79
WM18	0.03	0.06	na *	0.01	0.07	0.11	0.33	0.61

* not applicable.

**Table 11:** ILCR values determined for adults and children.

Sample ID	ILCR Adults				Total ILCR Adults	ILCR Children				Total ILCR Children
	Cu	Cr	Pb	Cd		Cu	Cr	Pb	Cd	
WM1	5.22 × 10^−3^	2.01 × 10^−4^	6.2 × 10^−7^	4.58 × 10^−4^	5.88 × 10^−3^	3.32 × 10^−2^	12.82 × 10^−4^	4 × 10^−6^	29.21 × 10^−4^	3.74 × 10^−2^
WM2	1.73 × 10^−2^	2.46 × 10^−4^	7.79 × 10^−6^	5.04 × 10^−3^	2.26 × 10^−2^	11.05 × 10^−2^	15.68 × 10^−4^	5.0 × 10^−5^	3.21 × 10^−2^	14.43 × 10^−2^
WM3	2.04 × 10^−2^	2.03 × 10^−4^	1.12 × 10^−5^	na *	2.06 × 10^−2^	13.00 × 10^−2^	12.95 × 10^−4^	7.2 × 10^−5^	na *	13.14 × 10^−2^
WM4	9.10 × 10^−3^	1.41 × 10^−4^	3.22 × 10^−5^	1.88 × 10^−4^	9.47 × 10^−3^	5.80 × 10^−2^	9.00 × 10^−4^	2.05 × 10^−4^	12.03 × 10^−4^	6.03 × 10^−2^
WM5	1.25 × 10^−2^	3.31 × 10^−4^	6.08 × 10^−6^	4.50 × 10^−3^	1.74 × 10^−2^	8.02 × 10^−2^	21.13 × 10^−4^	3.9 × 10^−5^	2.86 × 10^−2^	11.10 × 10^−2^
WM6	2.33 × 10^−2^	1.62 × 10^−3^	1.84 × 10^−5^	na *	2.49 × 10^−2^	14.85 × 10^−2^	1.03 × 10^−2^	1.18 × 10^−4^	na *	15.90 × 10^−2^
WM7	1.51 × 10^−2^	na *	na *	na *	1.51 × 10^−2^	9.67 × 10^−2^	na *	na *	na *	9.67 × 10^−2^
WM8	1.43 × 10^−2^	6.42 × 10^−6^	9.06 × 10^−6^	na *	1.43 × 10^−2^	9.12 × 10^−2^	4.1 × 10^−5^	5.8 × 10^−5^	na *	9.13 × 10^−2^
WM9	1.23 × 10^−2^	1.28 × 10^−5^	7.86 × 10^−6^	na *	1.23 × 10^−2^	7.83 × 10^−2^	8.2 × 10^−5^	5.0 × 10^−5^	na *	7.85 × 10^−2^
WM10	1.20 × 10^−2^	1.13 × 10^−4^	2.80 × 10^−6^	2.42 × 10^−3^	1.45 × 10^−2^	7.67 × 10^−2^	7.23 × 10^−4^	1.8 × 10^−5^	1.54 × 10^−2^	9.29 × 10^−2^
WM11	2.53 × 10^−2^	na *	na *	na *	2.53 × 10^−2^	16.13 × 10^−2^	na *	na *	na *	16.13 × 10^−2^
WM12	1.50 × 10^−2^	9.63 × 10^−5^	1.67 × 10^−5^	na *	1.52 × 10^−2^	9.61 × 10^−2^	6.14 × 10^−4^	1.07 × 10^−4^	na *	9.69 × 10^−2^
WM13	2.36 × 10^−2^	3.55 × 10^−4^	4.11 × 10^−6^	na *	2.39 × 10^−2^	15.04 × 10^−2^	22.63 × 10^−4^	2.6 × 10^−5^	na *	15.27 × 10^−2^
WM14	1.14 × 10^−2^	8.56 × 10^−5^	3.24 × 10^−6^	8.89 × 10^−4^	1.23 × 10^−2^	7.26 × 10^−2^	5.45 × 10^−4^	2.1 × 10^−5^	56.69 × 10^−4^	7.88 × 10^−2^
WM15	7.12 × 10^−3^	4.32 × 10^−4^	8.99 × 10^−6^	1.02 × 10^−3^	8.58 × 10^−3^	4.53 × 10^−2^	27.54 × 10^−4^	5.7 × 10^−5^	65.28 × 10^−4^	5.47 × 10^−2^
WM16	6.85 × 10^−3^	2.86 × 10^−4^	1.82 × 10^−6^	1.29 × 10^−3^	8.43 × 10^−3^	4.36 × 10^−2^	18.27 × 10^−4^	1.2 × 10^−5^	82.46 × 10^−4^	5.37 × 10^−2^
WM17	1.11 × 10^−2^	2.54 × 10^−4^	1.25 × 10^−5^	2.29 × 10^−2^	3.43 × 10^−2^	7.13 × 10^−2^	16.23 × 10^−4^	8.0 × 10^−5^	14.60 × 10^−2^	21.90 × 10^−2^
WM18	4.94 × 10^−3^	1.13 × 10^−4^	3.93 × 10^−6^	2.37 × 10^−3^	7.43 × 10^−3^	3.14 × 10^−2^	7.23 × 10^−4^	2.5 × 10^−5^	1.51 × 10^−2^	4.73 × 10^−2^

* na is “not applicable”.

## Data Availability

The original contributions presented in the study are included in the article/Supplementary Materials. Further inquiries can be directed to the corresponding authors.

## Supplementary Materials

The following supporting information can be downloaded at https://www.mdpi.com/article/10.3390/foods14203540/s1: Table S1. The ADI (mg kg^−1^ day^−1^) values determined for adults; Table S2. The ADI (mg kg^−1^ day^−1^) values determined for children.

## Abbreviations

Table following abbreviations are used in this manuscript:

EDXRF-FP	Energy Dispersive X-Ray Fluorescence Quantitative Analysis with Fundamental Parameters
NIST	National Institute of Standards and Technology
USEPA	United States Environmental Protection Agency
EPA	Environmental Protection Agency
HQ	Hazard quotients
HI	Hazard indices
EDI	Estimated daily intake
RfD	Reference dose
ILCR	Incremental lifetime cancer risk
ADI	Average daily intake
CSF	Cancer slope factor
DRI	Dietary reference intake
DMI	Daily metal intake
