# Supplementary material for: Assessment of the Impact of Metals in Wild Edible Mushrooms from Dambovita County, Romania, on Human Health
Source: Foods. 2025 Oct 17;14(20):3540. doi: 10.3390/foods14203540 (PMC12564050; doi:10.3390/foods14203540)
Supplement: Supplementary file 1 [file foods-14-03540-s001.zip › foods-3917852-supplementary.pdf]

Supplementary Data

Assessment of the Impact of Metals in Wild Edible Mushrooms from Dambovita County, Romania, on Human Health

Claudia Stihi [1,2]*, Crinela Dumitrescu [1]*

Table S1. The ADI (mg kg^{-1} day^{-1}) values determined for adults

Sample ID	ADI (mg kg^{-1} day^{-1})							
	Fe	Cu	Zn	Mn	Ni	Cr	Pb	Cd
WM1	0.027833	0.003073	0.037356	0.009497	0.000261	0.000402	0.000073	0.000073
WM2	0.121077	0.010208	0.034257	0.019290	0.019290	0.000492	0.000916	0.000800
WM3	0.295303	0.012005	0.062809	0.137067	0.000698	0.000407	0.001327	0.000000
WM4	0.116245	0.005359	0.073308	0.026266	0.000800	0.000282	0.003792	0.000030
WM5	0.029729	0.007409	0.036149	0.017745	0.000295	0.000663	0.000715	0.000715
WM6	0.069529	0.013717	0.027011	0.000000	0.000779	0.003253	0.002170	0.000000
WM7	0.128293	0.008937	0.060703	0.062270	0.000839	0.000000	0.000000	0.000000
WM8	0.096287	0.008423	0.103923	0.008637	0.000646	0.000013	0.001066	0.000000
WM9	0.144069	0.007237	0.107488	0.045954	0.000603	0.000026	0.000924	0.000000
WM10	0.087055	0.007088	0.034608	0.026566	0.000595	0.000227	0.000330	0.000385
WM11	0.135351	0.014894	0.022667	0.000000	0.000141	0.000000	0.000000	0.000000
WM12	0.117854	0.008881	0.023210	0.053872	0.000676	0.000193	0.001973	0.000000
WM13	0.132213	0.013889	0.049344	0.088147	0.000394	0.000710	0.000484	0.000000
WM14	0.071656	0.006707	0.111032	0.032567	0.000672	0.000171	0.000381	0.000141
WM15	0.233572	0.004190	0.048972	0.102523	0.000706	0.000865	0.001057	0.000163
WM16	0.037968	0.004032	0.021892	0.001010	0.002996	0.000574	0.000214	0.000205
WM17	0.379829	0.006587	0.035973	0.079227	0.000856	0.000509	0.001472	0.003638
WM18	0.027003	0.002906	0.042432	0.000000	0.000304	0.000227	0.000462	0.000377

* not applicable

Table S2. The ADI (mg kg^{-1} day^{-1}) values determined for children

Sample ID	ADI (mg kg^{-1} day^{-1})							
	Fe	Cu	Zn	Mn	Ni	Cr	Pb	Cd
WM1	0.177337	0.019580	0.238013	0.060512	0.001663	0.002563	0.000464	0.000464
WM2	0.771441	0.065039	0.218269	0.122906	0.122906	0.003136	0.005836	0.005099
WM3	1.881521	0.076492	0.400187	0.873322	0.004445	0.002591	0.008454	na*
WM4	0.740653	0.034142	0.467081	0.167356	0.005099	0.001800	0.024161	0.000191
WM5	0.189417	0.047204	0.230322	0.113061	0.001882	0.004227	0.004554	0.004554
WM6	0.443001	0.087400	0.172101	na*	0.004963	0.020725	0.013826	na*
WM7	0.817418	0.056940	0.386770	0.396751	0.005345	na*	na*	na*
WM8	0.613493	0.053667	0.662143	0.055031	0.004118	0.000082	0.006790	na*
WM9	0.917935	0.046114	0.684859	0.292798	0.003845	0.000164	0.005890	na*
WM10	0.554672	0.045159	0.220505	0.169265	0.003791	0.001445	0.002100	0.002454
WM11	0.862386	0.094900	0.144422	na*	0.000900	na*	na*	na*

WM12	0.750907	0.056585	0.147885	0.343247	0.004309	0.001227	0.012571	na*
WM13	0.842398	0.088491	0.314396	0.561626	0.002509	0.004527	0.003082	na*
WM14	0.456554	0.042732	0.707438	0.207497	0.004281	0.001091	0.002427	0.000900
WM15	1.488206	0.026697	0.312023	0.653226	0.004500	0.005509	0.006736	0.001036
WM16	0.241912	0.025688	0.139486	0.006436	0.019089	0.003654	0.001364	0.001309
WM18	0.172046	0.018516	0.270355	na*	0.001936	0.001445	0.002945	0.002400

* not applicable
